# Radiofrequency Ablation versus Resection for Colorectal Cancer Liver Metastases: A Meta-Analysis

**DOI:** 10.1371/journal.pone.0045493

**Published:** 2012-09-21

**Authors:** Mingzhe Weng, Yong Zhang, Di Zhou, Yong Yang, Zhaohui Tang, Mingning Zhao, Zhiwei Quan, Wei Gong

**Affiliations:** Department of General Surgery, Xinhua Hospital, School of Medicine, Shanghai Jiaotong University, Shanghai, China; H. Lee Moffitt Cancer Center & Research Institute, United States of America

## Abstract

**Background:**

No randomized controlled trial (RCT) has yet been performed to provide the evidence to clarify the therapeutic debate on liver resection (LR) and radiofrequency ablation (RFA) in treating colorectal liver metastases (CLM). The meta-analysis was performed to summarize the evidence mostly from retrospective clinical trials and to investigate the effect of LR and RFA.

**Methodology/Principal Findings:**

Systematic literature search of clinical studies was carried out to compare RFA and LR for CLM in Pubmed, Embase and the Cochrane Library Central databases. The meta-analysis was performed using risk ratio (RR) and random effect model, in which 95% confidence intervals (95% CI) for RR were calculated. Primary outcomes were the overall survival (OS) and disease-free survival (DFS) at 3 and 5 years plus mortality and morbidity. 1 prospective study and 12 retrospective studies were finally eligible for meta-analysis. LR was significantly superior to RFA in 3 -year OS (RR 1.377, 95% CI: 1.246–1.522); 5-year OS (RR: 1.474, 95%CI: 1.284–1.692); 3-year DFS (RR 1.735, 95% CI: 1.483–2.029) and 5-year DFS (RR 2.227, 95% CI: 1.823–2.720). The postoperative morbidity was higher in LR (RR: 2.495, 95% CI: 1.881–3.308), but no significant difference was found in mortality between LR and RFA. The data from the 3 subgroups (tumor<3 cm; solitary tumor; open surgery or laparoscopic approach) showed significantly better OS and DFS in patients who received surgical resection.

**Conclusions/Significances:**

Although multiple confounders exist in the clinical trials especially the bias in patient selection, LR was significantly superior to RFA in the treatment of CLM, even when conditions limited to tumor<3 cm, solitary tumor and open surgery or laparoscopic (lap) approach. Therefore, caution should be taken when treating CLM with RFA before more supportive evidences for RFA from RCTs are obtained.

## Introduction

Colorectal carcinoma (CRC) is the fourth most common malignancy worldwide [1], and raises serious concern in view of most cases developing metastases at presentation or during treatment. Liver as the only or initial metastatic site is found in 20% of the CRC patients [2]. Surgery is considered as the golden standard in the treatment of colorectal liver metastases (CLM), with 5-year overall survival rate ranging from 27% to 58% [3]–[5]. Nevertheless, only 10–25% of patients with CLM are eligible for surgical resection in terms of the extent and location of the disease and concurrent medical conditions [6].

Ablative therapeutic methods have been introduced as alternative measures to treat liver tumors such as cryoablation [7], percutaneous ethanol injection (PEI) [8], acetic acid injection [9], microwave coagulation [10], transcatheter arterialchemoembolization (TACE) [11] and radiofrequency ablation [12]. Among them, RFA is regarded as a promising and powerful technique for tumor destruction, and is recommended as the primary ablative therapy for CLM at most centers [13]. Nowadays, the RFA technology enables a single probe insertion to ablate a spherical zone exceeding 5 cm in diameter in vivo, which substantially expands its application in clinical practice [14].

The advantages of RFA treatment such as minimal invasiveness, better safety, equivalent local control and survival to liver resection (LR) have influenced the treatment strategy for hepatocellular carcinomas (HCC) and CLM [15]–[17]. Recently, two randomized clinical trials showed equivalent survival after percutaneous RFA and LR for HCC <5 cm [18], [19]. However, for those patients with CLM eligible for surgical treatment, whether RFA or LR is the better choice remains controversial. Two recent papers proposed a randomized trial comparing resection and radiofrequency ablation for resectable CLM [20]–[21]. We performed a meta-analysis of all the studies directly comparing LR and RFA in the treatment of CLM, preparing for the following RCTs.

## Materials and Methods

### Literature Search

QUOROM guidelines were followed for conducting meta-analysis. The study design and report were adhered to the PRISMA Statement guidelines (PRISMA S1). A systematic literature search was performed independently by two of the authors (WMZ and TZH) using Pubmed, Embase and the Cochrane Library Central at two different medical science information centers respectively affiliated to Fudan University and Shanghai Jiao Tong University. The search was limited to humans. No restriction was set for languages or date of publication. The search strategy was based on the following Medical Subject Heading terms (MeSH) and text words: “radiofrequency ablation”, “radio frequency ablation”, “resection”, “colorectal tumor”, “colorectal neoplasm”, “colorectal cancer”, “liver”, “metastases”, “metastasis”. The search was broadened by extensive cross-checking of the reference lists of all retrieved articles. When further information was required, the corresponding authors of relevant papers were contacted by the reviewers.

### Data Extraction

Data extraction was performed independently by the same investigators, and in the case of discrepancy, the decision was made by discussion with a third author (GW). The main extracted data included: (1) First author, the year of publication and the study type; (2) The number and characteristics of patients, (3) The outcome of the trials including the overall survival (OS) and disease-free survival (DFS) at 3 and 5 years plus mortality and morbidity.

### Inclusion Criteria

The following criteria were fulfilled for the studies included in the meta-analysis: (1) The studies comparing the original outcomes of RFA and LR in the treatment of colorectal cancer liver metastases; (2) The studies reporting at least 3- or 5-year overall survival; (3) If more than one studies were reported by the same institute or author, only the most recent or the highest level of studies were included.

### Exclusion Criteria

The following studies were excluded: (1) the original studies only assessing outcome of either RFA or LR; (2) those not using OS or DFS or with a follow up of less than 2 years; (3) those recruiting CLM patients treated with a combined therapy (LR+RFA); (4) review articles, letters, comments, case reports.

### Subgroup analysis

3 subgroups were evaluated: (1) the maximal tumor diameter was less than 3 cm; (2) solitary tumor; (3) RFA was conducted under laparoscopic or open surgery condition.

### Statistical Analysis

The meta-analysis was performed using Statistics/Data Analysis version 11.0 (Stata, Texas, USA). Calculation for dichotomous variables was carried out using the risk ratio (RR) and their 95% CI as the summary statistic. Owing to the between-study variability of sample size and detection methods, overall estimates were calculated by using the random effect model. Quantitative assessment of heterogeneity was explored by chi-square test with significance set at P value 0.10 and was measured using I-squared statistic. The potential for publication bias was graphically explored through the production of funnel plots, and tested for significance with Begg's test for asymmetry [22]. All statistical data were considered significant if the probability of a chance occurrence was less than 5% (p<0.05).

## Results

### Selection of trials

Of the 17 clinical trials initially met the inclusion criteria, 2 didn't display the specific comparison of the effects of RFA and LR [37], [38], 1 did not use 3- or 5-year OS [39] and 1 with no original data [40]. Finally, 13 studies [24]–[36] between 2003 and 2011 matched the selection criteria and were processed with meta-analysis (Table 1). Given the shortage of prospective randomized trials, 12 were retrospective studies and 1 was prospective study. 5 studies used percutaneous RFA [23], [25], [26], [32], [33], 2 studies used RFA during open surgery [24], [28], while the remaining 6 trials utilized RFA either via percutaneous or open surgery. Totally, 1886 subjects were involved in this meta-analysis, 1266 treated with LR and 620 treated with RFA. The pooled analysis of the patients' characteristics was as follows (LR vs. RFA): the mean male/female rate was 1.55 vs. 1.32; the mean age was 60.33 vs. 61.93; the mean tumor size and number were 3.35 cm vs. 2.52 cm and 1.28 vs. 1.38, respectively; the mean tumor stage α/β−χ rate and tumor node positive/negative rate were 0.21 vs. 0.31 and 1.23 vs. 1.11, respectively (Table 2, Table 3).

**Table 1:** Characteristics of included trials.

Author(year)	Reference	Design	Treatment	Number	M/F	Mean age(years)	Mean tumor size(cm)	Mean tumor number	Tumor stage1–2/3–4	tumor node status positive/negative
Oshowo	24	Retro	LR	20	10/10	63(52–77)	4(2–7)	1	-	-
(2003)			RFA Perc	25	11/14	57(34–80)	3(1–10)	1	-	-
Evrard	25	Retro	LR	17	10/7	57(25–88)	2.2	1(1–5)	-	-
(2004)			RFA Open	33	19/14	66(21–82)	1	3(1–8)	-	-
Abdalla	26	Retro	LR	190	-	60(23–88)	2.5	-	-	-
(2004)			RFA Open	57	-	60(23–88)	2.5	1(1–8)	-	-
White	27	Retro	LR	30	20/10	62(42–81)	2.7±1.1	1	-	17/13
(2007)			RFA Perc	22	8/15	62(48–77)	2.4±1.0	1	-	11/11
Gleisner	28	Retro	LR	192	121/71	61	3.5(2–5)	1–2.5	28/164	122/70
(2008)			RFA Perc	11	7/4	60	2.5(1.9–4)	1	0/11	7/4
Lee	29	Retro	LR	116	76/40	58(26–79)	<3 cm = 55.2% (64)>3 cm = 44.8% (52)	1	18/98	68/48
(2008)			RFA Open/Perc	37	11/26	59(28–75)	<3 cm = 73% (27)>3 cm = 27% (10)	1	11/26	20/17
Berber	30	Retro	LR	90	57/33	64	3.8±0.2	1	-	-
(2008)			RFA open	68	43/25	64	3.7±0.2	1	-	-
Hur	31	Retro	LR	42	27/15	-	2.6(0.6–8)	1	2/40	26/16
(2009)			RFA Open/Perc	25	15/10	-	2.5(0.8–3.6)	1	1/24	18/7
Reuter	32	Retro	LR	126	69/57	61.9	5.3	2.1	26/100	-
(2009)			RFA	66	46/20	63.5	3.2	2.8	14/52	-
McKay	33	Retro	LR	58	29/29	67(28–83)	4.1(1.5–14.5)	1(1–7)	-	-
(2009)			RFA Open/Perc	43	25/18	67(37–83)	3(1–7.5)	2(1–6)	-	-
Otto	34	Prosp	LR	82	49/33	62(38–80)	5(1–15)	2(1–11)	11/71	11/71
(2009)			RFA Perc	28	20/8	64(42–78)	2(1–5)	2(1–5)	4/24	22/60
Lee	35	Retro	LR	25	14/11	61(34–76)	4(0.7–9.7)	-	-	-
(2011)			RFA Perc	28	23/5	61(32–82)	2.05(1–4.8)	-	-	-
Kim	36	Retro	LR	278	168/110	57.1	2.6±2	1.5	-	-
(2011)			RFA Open/Perc	177	121/56	60.4	2.1±1	1.6	-	-

Retro: retrospective study. Prosp: prospective study not random. LR: hepatic resection. RFA: radiofrequency ablation. M: male. F: female. Perc: percutaneous. Open/Perc: contains data from both open and percutaneous surgery.

**Table 2:** Raw data of each included study.

Author(year)	Treatment	n	Overall Survival					Disease-free Survival					
			Hazard ratio	95% CI	P-value	3year OS	5year OS	Hazard ratio	95% CI	P-value	3year DFS	5year DFS	
Oshowo	LR	20	―	―	―	55%	42%	―	―	―	―	―	
(2003)	RFA (Perc)	25				53%	36%				―	―	
Evrard	LR	17	―	―	―	―	―	―	―	―	―	―	
(2004)	RFA (Open)	33				―	―				―	―	
Abdalla	LR	190	2.79	1.68–4.62	0.001	73%	58%	2.6	1.84–3.68	0.001	40%	31%	
(2004)	RFA Open	57				37%	21%				9%	7%	
White	LR	30	―	―	―	82%	57%	―	―	―	51%	36%	
(2007)	RFA Perc	22				84%	42%				0%	0%	
Gleisner	LR	192	1.77	0.75–4.21	0.01	72.00%	57.40%	1.41	0.59–3.35	0.01	41%	41%	
(2008)	RFA Perc	11				51.20%	28.30%				9%	0%	
Lee	LR	116	―	―	―	51%	66%	―	―	―	88%	85%	
(2008)	RFA Open/perc	37				32%	49%				53%	43%	
Berber	LR	90	1.24	0.91–1.66	0.16	70%	40%	―	―	―	45%	38%	
(2008)	RFA open	68				35%	30%				29%	0%	
Hur	LR	42	2.65	1.14–6.17	0.024	70%	50.10%	4.61	1.16–18.36	0.03	90%	89.70%	
(2009)	RFA Open/perc	25				60%	25.50%				76%	69.70%	
Otto	LR	82	1.035	0.478–2.239	0.93	60%	51%	0.523	0.304–0.901	0.017	40%	30%	
(2009)	RFA Perc	28				67%	48%				18%	18%	
Reuter	LR	126	―	―	―	55%	23%	―	―	―	42%	24%	
(2009)	RFA	66				42%	21%				24%	8%	
McKay	LR	58	2.78	1.43–5.26	0.02	60%	43%	―	―	―	―	―	
(2009)	RFA	43				39%	23%				―	―	
Lee	LR	25	1.74	1.37–2.21	0.001	68%	44.00%	1.4	1.12–1.75	0.004	40%	12%	
(2011)	RFA Perc	28				35.70%	17.90%				10.70%	0%	
Kim	LR	278	3.613	1.422–9.193	0.007	59%	44.60%	3.821	1.518–9.62	0.004	32%	28%	
(2011)	RFA	177				49.70%	35.60%				26%	20.30%	

LR: liver resection. RFA: radiofrequency ablation. CI: confidence interval. Perc: percutaneous. Open/Perc: contains data from both open and percutaneous surgery.

**Table 3:** Summary of patient's characteristics.

Characteristics	LR	N_LR_	RFA	N_RFA_
M/F	1.55±0.26	1076	1.32±0.56	563
age	60.33±2.54	1224	61.93±2.55	595
Mean tumor size(cm)	3.35±0.98	1150	2.52±0.67	583
Mean tumor number	1.28±0.21	1051	1.38±0.42	535
Tumor stage I–II/III–IV rate	0.21±0.05	558	0.31±0.10	167
tumor node status positive/negative rate	1.23±0.68	462	1.11±0.96	123

LR: liver resection. RFA: radiofrequency ablation. M: male. F: female.

### Overall survival

**Figure 1:**
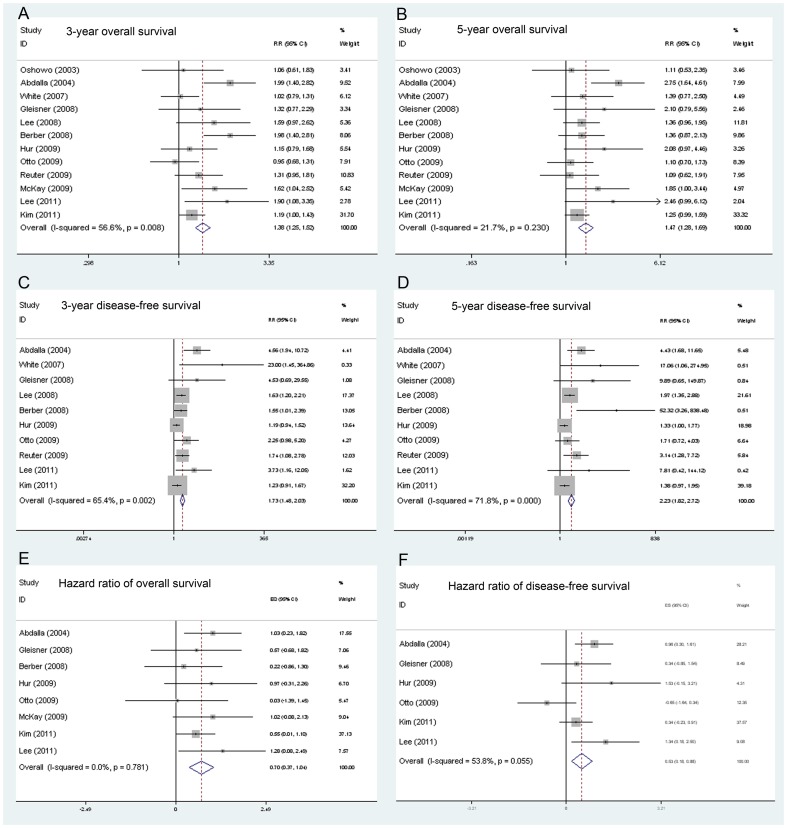
Results of the meta-analysis on overall survival, disease-free survival, overall survival hazard ratio and disease-free survival hazard ratio. A. Results of the meta-analysis on overall survival at 3 years. B. Results of the meta-analysis on overall survival at 5 years. C. Results of the meta-analysis on disease-free survival at 3 years. D. Results of the meta-analysis on disease-free survival at 5 years. E. Results of the meta-analysis on overall survival hazard ratio. F. Results of the meta-analysis on disease-free survival hazard ratio.

The statistic data was significantly favorable to LR group at 3-year survival (11 trials reported the data, RR: 1.377, 95% CI: 1.246–1.522) and 5-year survival (11 trials reported the data, RR: 1.474, 95%CI: 1.284–1.692) (Figure 1). Moreover, stratified meta-analysis showed the LR group had better long-term survival than RFA group in all 3 subgroups (Table 4).

**Table 4:** Results of the meta-analysis for LR vs RFA in treatment of CLM.

Variables	Time interval	Subgroups	Nsuvival/N_LR_	Nsuvival/N_RFA_	LR vs RFARR (95%CI)	p	I^2^	Ref.
Overall survival	3 years	Total	802/1249	269/587	1.377(1.246–1.522)	<0.001	56.6%	26–34
		<3 cm	157/213	33/72	1.680(1.279–2.208)	<0.001	90.0%	26,31
		Solitary	306/481	150/290	1.263(1.109–1.439)	<0.001	64.0%	24,27,29–31,36
		Open	139/280	29/125	2.549(1.801–3.609)	<0.001	73.4%	26,30
		Perc	240/349	55/114	1.143(0.947–1.379)	0.014	48.8%	24,27,28,34,35
	5 years	Total	610/1249	182/587	1.474(1.284–1.692)	<0.001	21.7%	26–36
		<3 cm	123/213	20/72	2.168(1.442–3.260)	<0.001	84.4%	26,31
		Solitary	250/481	119/290	1.209(1.025–1.426)	0.024	0.0%	24,27,29–31,36
		Open	95/280	24/125	2.012(1.321–3.064)	0.001	81.8%	26,30
		Perc	188/349	39/114	1.426(1.062–1.915)	0.018	0%	24,27,28,34,35
Disease-free survival	3 years	Total	539/1171	135/519	1.735(1.483–2.029)	<0.001	65.4%	26–32,34–36
		<3 cm	98/213	18/72	2.238(1.480–3.385)	<0.001	97.7%	26,31
		Solitary	343/653	101/276	1.435(1.212–1.699)	<0.001	61.5%	27–31,36
		Open	117/280	25/125	2.309(1.544–3.453)	<0.001	82.5%	26,30
		Perc	137/329	9/114	3.853(2.065–7.190)	<0.001	6.6%	27,28,34,35
	5 years	Total	456/1171	83/519	2.227(1.823–2.720)	<0.001	71.8%	26–32,34–36
		<3 cm	81/213	17/72	1.104(1.039–1.173)	0.001	97.9%	26,31
		Solitary	324/653	64/276	2.014(1.624–2.499)	<0.001	78.8%	27–31,36
		Open	93/280	4/125	8.477(3.565–20.156	<0.001	70.5%	26,30
		Perc	118/329	5/114	3.763(1.762–8.033)	0.001	41.3%	27,28,34,35

LR: liver resection. RFA: radiofrequency ablation. RR: risk ratio. CI: confidence interval. <3 cm refers to the maximal tumor diameter was less than 3 cm. Open means that RFA was conducted under open surgery condition. Perc means that RFA was conducted percutaneously.

### Disease-free survival

As Table 3 shows, the 3-year DFS (RR 1.735, 95% CI: 1.483–3.385) and 5-year DFS (RR 2.227, 95% CI: 1.823–2.720) was significantly higher in the LR group (Figure 1). The significantly higher DFS rates in LR group were also observed in all 3 subgroups.

### Safety

The postoperative morbidity was significantly higher in the LR group than in the RFA group. (9 trials reported the data, RR: 2.495, 95% CI: 1.881–3.308). However, no difference was observed in terms of postoperative mortality (8 trials involved, RR: 1.391, 95% CI: 0.306–6.326) (Table 5). The mean length of hospital stay was 11.02±0.11 days for LR group and 4.05±0.10 days for RFA (standardized mean difference: 3.284, 95% CI: 3.052–3.516, P<0.001).

**Table 5:** Meta-analysis of the safety of liver resection and radiofrequency ablation.

Variables	LR	N_Morbidity_/N_LR_	RFA	N_Motality_/N_RFA_	RR (95%CI)	p	I^2^	Reference
Morbidity	24.10%	220/913	9.98%	47/471	2.495(1.881–3.308)	0.009	60.70%	22,26,28–34
Mortality	0.31%	2/639	0.34%	1/294	1.391(0.306–6.326)	0.407	0.0%	22,26,28–32

LR: liver resection. RFA: radiofrequency ablation.

### Publication Bias

**Figure 2:**
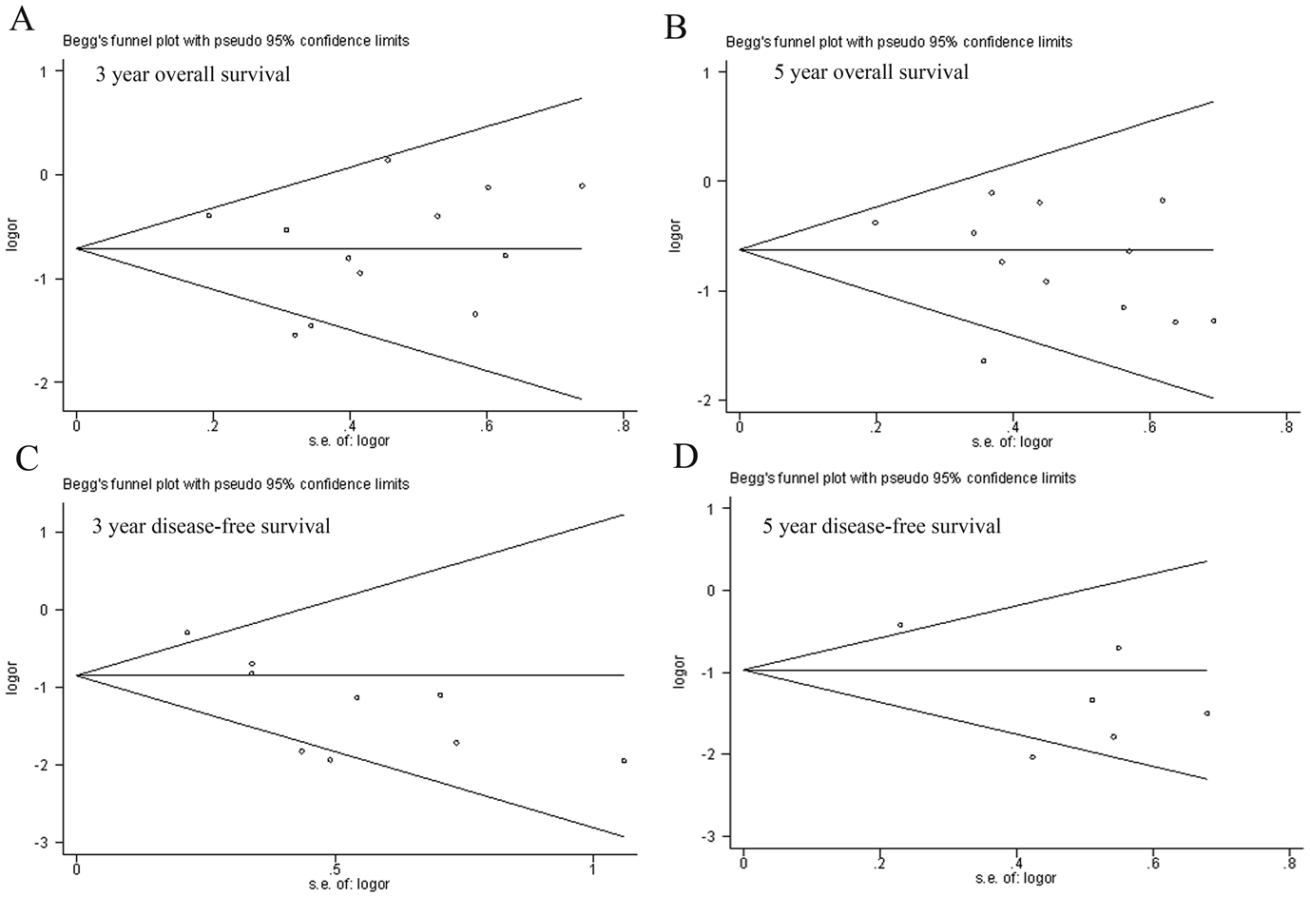
Funnel plot to detect publication bias. A. Begg's test result of 3-year survival. B. Begg's test result of 5-year survival. C. Begg's test result of 3-year disease-free survival. D. Begg's test result of 5-year disease-free survival.

The funnel plot did not show evidence of publication bias by Begg's test in 3-year survival (z = 0.41, Pr>|z| = 0.732, continuity corrected), 5-year survival (z = −1.51, Pr>|z| = 0.15, continuity corrected), 3-year DFS (z = −1.25, Pr>|z| = 0.251, continuity corrected), and 5-year DFS(z = −0.19, Pr>|z| = 1.0, continuity corrected) (Figure 2).

## Discussion

Surgical resection currently is the gold standard in the treatment of resectable colorectal cancer liver metastases [41]. 5-year survival in resected patients was about 25% versus 0% for the untreated in some early retrospective studies [42]. It has been reported recently that improved surgical techniques brought the 5-year survival rates up to 30–35% [41]. However, traditional hepatectomy is being challenged by a number of ablative techniques, for instance, microwave ablation, laser ablation, cryoablation and radiofrequency ablation which allow a wide varialblity in the reported 5-year survival rate (14–55%) for the unresectalbe colorectal liver metastases [6]–[12].

Two recently published randomized clinical trials (RCTs) showed equivalent survival rate of percutaneous RFA to surgical resection for hepatocellular caricinomas (HCC) <5 cm. While RCTs for CLM patients are pending, there is a rising demand for comparing as much evidence as possible to clarify whether RFA or LR is better in the treatment of CLM [18], [19]. We performed this meta-analysis which showed that in the treatment of CLM, LR was superior to RFA. LR had a significant higher survival at 3 and 5 years as well as disease-free survival at 3 and 5 years. It was reported that better prognosis was achieved after RFA when maximal size of the tumors was less than 3 cm as consequence of the disease free margins [43] which the authors claimed that the tumor should not exceed 3.5 cm in longest axis to obtain a safety margin of 1 cm all around the lesion [44]. However, in our subgroup of tumor size<3 cm, the data did not show favorable outcomes.

It was also reported that a significant difference in the number of patients with solitary tumor between the LR and RFA leaded to the prognostic inequality [24], [27], [29]–[31], [36]. However, we found that patients with solitary tumor had higher OS and DFS after LR. Moreover, two studies showed lower local recurrence rates for open surgical approach comparing with percutaneous approach in RFA [45], [46], and this meta-analysis demonstrated that even the open surgical ablation group was still unable to match the survival of LR. It is suggested that although tumor<3 cm, solitary tumor and open surgery or laparoscopic (lap) approach are the prognostic factors favorable to RFA, performing RFA in such scenarios still cannot achieve a comparable OS and DFS to those of LR.

It could be explained in several aspects. Firstly, in the retrospective studies, “unresectable” CLM as a main indication for RFA may lead to inevitable selection bias. “Unresectable” patients refer to those who would not be tolerant to surgery because of poor healthy condition, inadequate functional reserve of the remaining liver or special locations of the metastatic tumor such as bilobes of the liver or proximity to large vessels. Secondly, the resection allows better intraoperative staging and postoperative pathological evaluation, which helps make an optimized postoperative treatment strategy of chemotherapy and biotherapy. Thirdly, an estimated 0–1.4% risk of electrode track seeding was reported to occur after percutaneous RFA, leaving the possibility of distant recurrence [47].

On the other hand, we should not neglect the non-oncological advantages of RFA over hepatic resection, such as lower complication rate (18.3% vs 3.9%, p<0.01), and shorter hospital stay (9.2±0.6 vs 3.9±0.4, p<0.01). Most patients undergoing percutaneous RFA only require an overnight stay, while elderly patients stay 2–3 days [48]. For laparoscopic and open RFA, the mean hospital day is 1–3 days and 4–7 days respectively [49]. RFA has a big advantage over the LR group with a mean hospital stay of 12.5 days.

Shortly after we finished our meta-analysis, a similar paper which focused on solitary CLM was recently published by Wu et al [50]. Consistent with our findings, they found that LR group had better 5-year survival rate and comparable postoperative mortality comparing with RFA group. However, in contrast to their result that two groups had no difference in terms of postoperative morbidity, our study found that the postoperative morbidity was significantly higher in the LR group than in the RFA group. This might result from different data we adopted in our study in which we examined all the colorectal liver metastasis including multiple liver metastasis.

The only way to balance the selection bias and consequently find out whether RFA can reach equal outcome is to hold a randomized controlled trial. Mulier et al [51] proposed a randomized trial of RFA versus resection for resectable colorectal liver metastases with the following inclusion criteria: resectable CLM; no contraindication for RFA; only small tumors (<3 cm); RFA only by open surgical approach; only tumors away from large vessels unless a Pringle maneuver can be safely applied; RFA only by experienced physicians; intentional margin of 1 cm; only with electrodes that produce a well-documented, regular and predictable ablation zone. It is expected that RCT can provide higher level evidence for the utility of RFA and pave the way for the future application of RFA in the treatment of resectable CLM.

Liveraghi et al [52] proposed to conduct RFA ablation during the interval between diagnosis and resection as a “test-of-time” therapeutic option. The patients whose lesions were treated adequately after RFA ablation may avoid surgical resection and if it was found tumor residence or local recurrence after RFA, surgical resection was then processed. 88 consecutive patients with 134 colorectal carcinoma liver metastases who were potential candidates for hepatic metastasectomy were undergone RFA ablation. Among the 53 patients who achieved complete tumor ablation after RFA, 52 (98%) were spared surgical resection; 23 (44%) remained free of disease, 29 (56%) developed disease progression and no patient who had been treated with RFA ablation became unresectable due to the growth of metastases. It provides a novel way of RFA as the first-line therapy that can avoid unnecessary surgery.

## Conclusions

Since currently no RCT data are available for treating CLM patients, the vast majority of studies included in this meta-analysis comparing the effect of RFA and LR are retrospective. Liver resection provided superior OS and DFS over RFA, even when performed on tumor<3 cm or solitary tumor, or using open/lap approach. Conversely, RFA shows advantage over surgical resection in morbidity and length of hospital stay. Due to a lower OS and DFS after RFA suggested by the meta-analysis, caution should be taken when treating CLM with RFA before more supportive evidence for RFA treatment are obtained from RCTs.

## Supporting Information

Flow of Included Studies S1 **The flow diagram depicts the flow of information through the different phases of our systematic review.** It maps out the records identified, included and excluded, and the reasons for exclusions.
(DOC)

PRISMA S1 **The PRISMA checklist contains items pertain to the content of reviewed papers which include the title, abstract, methods, results, discussion and funding.**
(DOC)
