# Supplementary material for: Radiofrequency Ablation versus Resection for Colorectal Cancer Liver Metastases: A Meta-Analysis
Source: PLoS One. 2012 Sep 21;7(9):e45493. doi: 10.1371/journal.pone.0045493 (PMC3448670; doi:10.1371/journal.pone.0045493)
Supplement: Flow of Included Studies S1 — The flow diagram depicts the flow of information through the different phases of our systematic review. It maps out the records identified, included and excluded, and the reasons for exclusions. (DOC) [file pone.0045493.s001.doc]

**Flow of Included Studies**

Literatures identified through electronic searches of databases

*n*=120

Excluded n=90

Irrelevant studies excluded by browsing abstract and title

Studies selected based on

abstract and title

n=30

Excluded n=14

Reviews n=13

Meta-analysis n=1

Full text analysis

n=16

Excluded n=4

No special comparison n=2

No critical data (3,5-year overall survival) n=1

No original data n=1

Fulfill the criteria and included in meta-analysis n=12
